# Cost Comparison of Treatment Alternatives for Pleural Effusion and Ascites from a Payer Perspective: Are There Cost Savings from Indwelling Catheters?

**DOI:** 10.1089/jpm.2022.0592

**Published:** 2023-11-08

**Authors:** Ann-Cathrine Siefen, Leonie Eilers, Christoph T. Baltin, Florian Kron

**Affiliations:** ^1^ VITIS Healthcare Group, Cologne, Germany.; ^2^ Department I of Internal Medicine, University of Cologne, Faculty of Medicine and University Hospital Cologne, Cologne, Germany.; ^3^ Faculty of Medicine and University Hospital Cologne, Centre for Integrated Oncology (CIO ABCD), University of Cologne, Cologne, Germany.; ^4^ KCM KompetenzCentrum für Medizinoekonomie, FOM University of Applied Sciences, Essen, Germany.; ^5^ Department of Orthopedics and Trauma Surgery, Faculty of Medicine and University Hospital of Cologne, University of Cologne, Cologne, Germany.

**Keywords:** ascites, cost comparison, indwelling catheter system, pleural effusion

## Abstract

**Background:**

The presence of pleural effusions and ascites in patients is often considered a marker of illness severity and a poor prognostic indicator. This study aims to compare inpatient and outpatient costs of alternative invasive treatments for ascites and pleural effusions.

**Methods:**

The retrospective single-institution study included inpatient cases treated for pleural effusion (J90 and J91) or ascites (R18) at the University Hospital Cologne (UHC) in Germany between January 01, 2020, and December 31, 2021. Costs for punctures and indwelling catheter systems (ICSs) as well as pleurodesis were analyzed in different comparator treatment pathways. Real-world data from the UHC tertiary care center were based on diagnosis-related group fees from 2020 to 2021. A simulation of outpatient expenses was carried out to compare inpatient and outpatient costs for each pathway from a payer perspective.

**Results:**

A total of 4323 cases (3396 pleural effusions and 1302 ascites) were analyzed. For ascites, inpatient implantation with home care drainage was found to be the most expensive option, with total costs of €1,918.58 per procedure, whereas outpatient puncture was the least expensive option at €60.02. For pleural effusions, the most expensive treatment pathway was pleurodesis at €8,867.84 compared with the least costly option of outpatient puncture resulting in total costs per procedure of €70.03. A break-even analysis showed that outpatient puncture remains the most inexpensive treatment option, and the ICS comprises a cost-saving potential. Longevity of several months with the use of ICSs results in both enhanced quality of life for patients and increased cost savings.

## Introduction

Patients with cancer usually battle multiple side diagnoses, in addition to their main diagnosis, worsening their quality of life. In particular, the presence of pleural effusions and ascites is considered a marker of illness severity and a poor prognostic indicator.^1,2^ Given new oncological treatments prolonging the survival of patients, underlying health care costs for the treatment of pleural effusions and ascites become even more important.

Ascites is the pathological accumulation of fluid in the free peritoneal space. In general, it is triggered by fluid leakage from blood vessels into the abdominal cavity. Symptoms include abdominal pain, increased abdominal girth, nausea and vomiting, shortness of breath, and fatigue. Ascites is most often associated with cirrhosis of the liver, followed by heart failure, kidney failure, infection, or cancer. Malignant ascites account for ∼10% of all ascites cases and life expectancy is ∼20 weeks from its diagnosis.^2–4^ Moreover, one study suggests that patients with liver disease were twofold more likely to die in an institution with 15% higher costs, compared with those without liver disease.^5^

A pleural effusion is the pathological increase of fluid in the pleural space, and the effusion can be either benign or malignant. Congestive heart failure, pleural infection, and malignancy are the leading causes of pleural effusions.^6^ Approximately every second cancer patient develops malignant pleural effusion.^7^ The most common symptoms are shortness of breath, irritable cough, and chest pain; however, the disease can also be asymptomatic. Median life expectancy ranges from 3 to 12 months for patients with malignant pleural effusion following a palliative treatment strategy alleviating secondary symptoms.^8^ For nonmalignant pleural effusions, patients have a longer median life expectancy of multiple years.^1,9^

So far, an effective antitumor therapy for malignant pleural effusion and malignant ascites due to peritoneal carcinomatosis does not exist. Instead, treatment is most often limited to palliation of symptoms, including dyspnea, nausea, chest, abdominal pain, and anorexia.^10^ Current treatment options aim to prevent recurrences involving diuresis, punctures/paracentesis, shunts, local chemotherapy, and permanent drainage or indwelling catheter systems (ICSs). For ascites, treatment options using an abdominal water pump or transjugular intrahepatic portosystemic shunt are also relevant.

Additional treatment options for pleural effusion include chemical pleurodesis and pleurectomy. However, recent studies show that ICS is more effective and more appropriate for symptom control and improvement of quality of life compared with pleurodesis.^11–13^ Following an outpatient therapy strategy, the ICS enables patients to stay in their home environment.

These procedures are often initialized under local anesthesia as minimally invasive procedures resulting in less pain compared with other forms of therapy, for example, chemical pleurodesis. Furthermore, these procedures also minimize the risk of injury to internal organs and subsequently lower the infection rate.^14^ Additionally, patients with pleural effusions have an increased chance of spontaneous pleurodesis.^15,16^

Although numerous studies have evaluated potential benefits for patients, there is a lack of health economic studies analyzing the implications of current treatment options from an insurer's perspective.^17^ The cost-effectiveness is also shown to depend on various factors such as complications, duration, and severity of the underlying diseases, as well as survival. For malignant ascites, the National Institute for Health and Care Excellence (NICE) anticipated in their Medical Technologies Evaluation Programme (MTEP) savings to the National Health Service (NHS) of the United Kingdom when using peritoneal drainage compared with repeated large-volume paracenteses in an inpatient setting.^18^

The study by Bohn and Ray similarly suggested that a peritoneal catheter will be economically beneficial if patients require large-volume paracentesis more than nine times or if patients drain less than 5 L of fluid per procedure.^19^ In addition, Wu et al. identified tunneled peritoneal catheters to be cost-effective in comparison with large-volume paracentesis due to the reduced risk of infection, emergency department visits, and length of stay (LOS).^20^

A systematic literature review by Botana-Rial et al. found that the cost of the indwelling pleural system ranges from €1,100 (for patients surviving less than six weeks) to more than €5,000 in case of complications.^21^ Compared with pleurodesis, the cost of the ICS was significantly lower only when patient survival was less than 14 weeks or when no home care was required.

The purpose of this study was to assess and compare the costs of treatment alternatives for pleural effusion and ascites patients from a payer perspective in Germany. A primary focus lies on the health economic effects of ICS in terms of cost savings.

## Methods

### Data extraction

This study is a retrospective single-institution analysis of inpatient cases treated at the University Hospital Cologne (UHC) in Germany between January 01, 2020, and December 31, 2021. According to the International Classification of Diseases, 10th revision, German Modification, (ICD-10-GM), the underlying cases were precoded with either pleural effusion (J90 and J91) or ascites (R18) as the main or secondary diagnosis.

### Treatment alternatives/comparison groups

As described previously, there are several therapy options for both diseases. For ascites, punctures and ICSs are most commonly described in the literature.^14,17,19,20^ For pleural effusions, this applies to pleurodesis as well as punctures and ICSs.^11–13,21^

#### Ascites

**FIG. 1.**
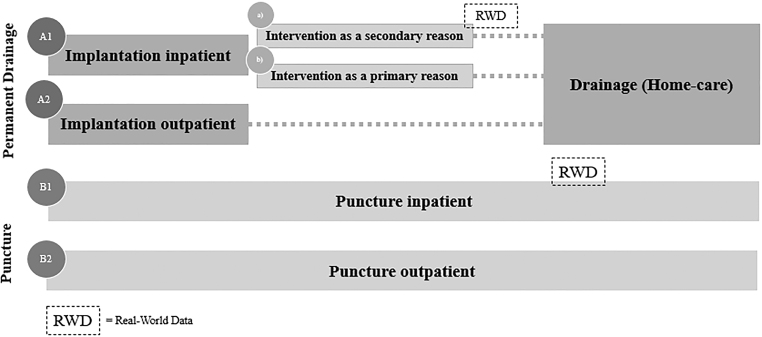
Treatment pathways (ascites).

For ascites, five different comparator treatment pathways have been defined, that is, A1a, A1b, A2, B1, and B2 (compare Fig. 1). Along each pathway, the occurring costs were analyzed. As the pathways include not only inpatient but also outpatient treatments, the cost data of the university hospital had to be supplemented. For this purpose, we modeled the outpatient expenses. The pathways can be subdivided into punctures and ICSs. The ICS initially needs to be implanted before the patient can drain the fluid at home by themselves or by a caretaker.

For ascites patients, the ICS implantation can be conducted in an in- or outpatient setting (pathway A1 vs. A2). For the A1 (implantation inpatient) pathway, the implantation can, on the one hand, be the main (primary) cause of hospitalization (A1b). On the other hand, ICS only reflects a secondary treatment during their hospital stay if, for example, another surgery (e.g., cardiothoracic surgery) is the primary cause of hospitalization and ICS is implemented as a result (A1a). Regarding patients who receive a puncture, two groups can be differentiated: outpatient (B1) or inpatient puncture (B2).

#### Pleural effusion

**FIG. 2.**
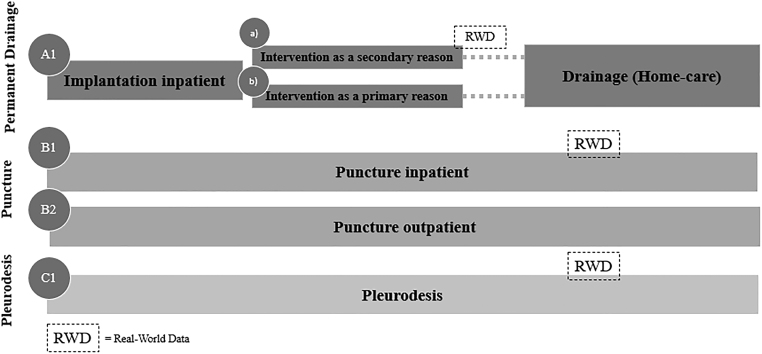
Treatment pathways (pleural effusion).

Looking at the comparator pathways for pleural effusion, the groups are analogous to the ascites comparator pathways (Figs. 1 and 2). One exception is that ICS implantations for pleural effusions are typically not performed in an outpatient setting in Germany, which is underlined by the absence of a respective outpatient procedure code and in return the lack of reimbursement. Therefore, they are not part of the analysis. Additionally, pleurodesis as a third relevant treatment option for pleural effusion is included.

The objective of our analysis is to quantify the costs for both treatment pathways (ascites and pleural effusion) from a payer perspective.

### Database

#### Inpatient sector

For inpatient costs, our cost assessment is based on real-world data (RWD) of the UHC. Patients under the age of 20 were excluded. The database includes information on patient characteristics, main and secondary diagnoses, German diagnosis-related group (DRG) allocation and revenue, German procedure classification (Operationen- und Prozedurenschlüssel or *OPS*), LOS, and nursing degrees.

The aim was to identify patients who represent the (partial) inpatient treatment pathways. Therefore, we searched the database for the following OPS codes and LOS values:

- for pathway A1 (inpatient ICS implantation),
  ○ LOS: 1–2 days and
  ○ OPS codes
    ▪ 5–549.2 or 8–148.0 for ascites drainage and
    ▪ 8–144.0, 8–144.1, or 8–144.2 for pleurodesis drainage.
- for pathway B1 (inpatient puncture),
  ○ LOS: 1 day and
  ○ *OPS* codes:
    ▪ 1–853.2 and 8–153 for ascites puncture and
    ▪ 1–844 and 8–152.1 for pleural effusion puncture.
- for pathway C1 (pleurodesis),
  ○ LOS: any LOS and
  ○ *OPS* codes: 5–345.1 - 5–345.6.

Based on the median DRG fees of each subgroup, the costs for the comparator pathways were calculated. To validate the median DRG costs resulting from our RWD analysis, we cross-checked the resulting DRG using a web-based grouper tool.^22^ In case the cross-check resulted in a DRG with a significantly lower fee, we analyzed and calculated costs for all cases with this respective DRG in the RWD.

#### Outpatient sector

Costs that occur in the outpatient sector have been modeled, that is, for

- pathway A2 (outpatient implantation in the ascites group) and
- pathway B2 (outpatient puncture in ascites and pleural effusion groups).

Costing data are taken from the so-called German Uniform Assessment Standard (einheitlicher Bewertungsmaßstab or EBM), which is the basis for remuneration of outpatient services. Outpatient treatments are documented and reimbursed based on *EBM* codes for each procedure performed. In contrast to inpatient *OPS* codes (which indirectly lead to a certain DRG), the *EBM* codes are directly associated with a fee.

In addition, costs for nursing services were taken from the German schedule of fees for care services. Material costs for an ICS are given by the list price *(Lauer-Taxe*) of the ASEPT ICS. ASEPT is an example of a permanent drainage system, which was approved by the FDA in 2015.^23^

### Cost components per pathway

The analysis was conducted from a payer perspective, that is, the German statutory health insurance (SHI). For the inpatient sector, costs of all types are included in the DRG fees. One exception, at least for the German DRG system, is the remuneration for care services that are reimbursed separately. Costs incurred within the inpatient pathways are therefore included using the cost data from the UHC, which are based on DRGs.

Based on the RWD, costs incurred by patients with versus without neoplasms have been calculated to assess whether these subgroups incur substantially diverging costs.

Outpatient cost components, in contrast, are not merged into one single fee, but consist of a variety of different positions that are summed up. We therefore modeled the inpatient pathways by identifying all procedures undertaken for the respective treatment as well as the associated *EBM* codes. This included the following components: quarterly fees (lump sum), *EBM* positions for treatments and ambulatory surgeries, diagnostics, and material costs. Costs for the ICS as well as home care services were added as they are not represented by the *EBM*.

When comparing the home care treatment pathways (including the ICS) with those pathways consisting of regular physician visits (in- or outpatient), the latter options incur not only medical costs but also transportation costs, in particular, for patients with high care needs. Therefore, we aimed to approximate this additional cost component by making the following assumptions: all patients who are dependent on care services (degrees 3–5 based on the German classification system; 5 being the highest degree) will need and be eligible to receive dedicated patient transport. We determined the share of patients who fall into one of these categories based on our RWD from the UHC.

To assess the costs occurring per pathway, each cost component was analyzed regarding frequency. Finally, after analyzing and simulating the total costs per pathway, we compared each pathway in a break-even analysis, emphasizing the costs per treatment duration in weeks. Therefore, we set up the following cost functions:


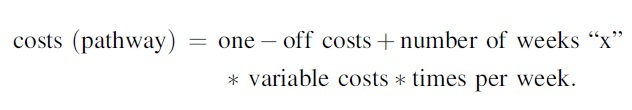


All costs are given in euros (€). We calculated median costs. The RWD from UHC are based on DRG fees from 2020 and 2021. All other costs reflect the 2021 prices.

All descriptive statistics have been assessed using Microsoft Excel (MS 365 Business Standard).

## Results

### Patient characteristics

In total, 4323 ascites and pleural effusion cases have been treated in the UHC from 2020 to 2021. The majority are pleural effusion cases (3396), whereas ascites accounts for 1302 cases. Three hundred seventy-five cases were based on both diagnoses. Across all 4323 cases, the 3 most common main diagnoses can be assigned to the following ICD-10 chapters: (IX.) “Diseases of the circulatory system” (37%), (II) “Neoplasms” (23%), and (XI.) “Diseases of the digestive system” (11%).

Because one patient may have more than one case (e.g., because they have several stays throughout the years), this reflects 3690 individual patients, of which 3494 were eligible (i.e., older than 19 years). Of all eligible patients, 63% were male. Most patients (29%) were between 70 and 79 years old, followed by those between 60 and 69 years (25%). Table 1 displays detailed case and patient characteristics.

**Table 1.** Case and Patient Characteristics (Real-World Data)

Characteristics	*n*	%
Cases		
All hospital cases	4323	100
Ascites	1302	30.12
Pleural effusion	3396	78.56
-/- Ascites and pleural effusion	375	8.67
ICD-10 chapters of main diagnoses		
(I) Certain infectious and parasitic diseases; A00–B99	86	1.99
(II) Neoplasms; C00–D48	992	22.95
(III) Diseases of the blood and blood-forming organs and certain disorders involving the immune mechanism; D50–D89	14	0.32
(IV) Endocrine, nutritional and metabolic diseases; E00–E90	16	0.37
(V) Mental and behavioural disorders; F00–F99	0	0.00
(VI) Diseases of the nervous system; G00–G99	47	1.09
(VII) Diseases of the eye and adnexa; H00–H59	0	0.00
(VIII) Diseases of the ear and mastoid process; H60–H95	0	0.00
(IX) Diseases of the circulatory system; I00–I99	1604	37.10
(X) Diseases of the respiratory system; J00–J99	271	6.27
(XI) Diseases of the digestive system; K00–K93	486	11.24
(XII) Diseases of the skin and subcutaneous tissue; L00–L99	21	0.49
(XIII) Diseases of the musculoskeletal system and connective tissue; M00–M99	77	1.78
(XIV) Diseases of the genitourinary system; N00–N99	116	2.68
(XV) Pregnancy, childbirth and the puerperium; O00–O99	4	0.09
(XVI) Certain conditions originating in the perinatal period; P00–P96	42	0.97
(XVII) Congenital malformations, deformations, and chromosomal abnormalities; Q00–Q99	104	2.41
(XVIII) Symptoms, signs and abnormal clinical and laboratory findings, not elsewhere classified; R00–R99	205	4.74
(XIX) Injury, poisoning and certain other consequences of external causes; S00–T98	233	5.39
(XXII) Codes for special purposes; U00–U99	4	0.09
(XX) External causes of morbidity and mortality; V01–Y98	0	0.00
(XXI) Factors influencing health status and contact with health services; Z00–Z99	1	0.02
Individual patients		
All patients	3690	
Eligible patients (≥20 years old)	3494	100
Sex		
Male	2189	62.65
Female	1304	37.32
Other	1	0.03
Age (years)		
20–29	55	1.57
30–39	124	3.55
40–49	192	5.50
50–59	573	16.40
60–69	873	24.99
70–79	1002	28.68
80–89	605	17.32
90–99	70	2.00

### Cost analysis

Table 2 shows the DRG costs on a case basis, including sample sizes per subgroup. Cases with neoplasms do not incur higher or lower costs than cases without neoplasms. Therefore, no distinction was made in the following calculations.

**Table 2.** Diagnosis-Related Group Costs (Real-World Data Per Inpatient Pathway and Subgroup)

	Overall			Ascites (R18)			Pleural effusion (J90 and J91)		
	*n*	Mean DRG costs	Median DRG costs	*n*	Mean DRG costs	Median DRG costs	*n*	Mean DRG costs	Median DRG costs
All cases	4323	€19,428.42	€13,208.30	1302	€16,565.65	€5,271.36	3396	€21,403.73	€14,735.49
Eligible cases (≥20 years old)	4117	€18,264.14	€12,692.38	1175	€12,642.51	€4,669.45	3284	€20,957.98	€14,520.53
Drainage, LOS 1 or 2 days	54	€3,248.04	€3,274.97	21	€3,419.71	(€3,286.19)	33	€3,138.80	(€3,263.75)
Thereof with C00–D48 diagnosis	40	€3,367.50	€3,028.23	17	€3,481.91	€2,792.70	23	€3,282.93	€3,263.75
Thereof without C00–D48 diagnosis	14	€2,906.72	€3,529.19	4	€3,155.34	€3,542.28	10	€2,807.28	€3,338.53
Puncture, LOS 1 day	138	€1,027.02	€721.54	116	€1,060.36	**€721.54**	22	€851.20	**€833.70**
Thereof with C00–D48 diagnosis	79	€1,025.40	€721.54	65	€1,060.83	€721.54	14	€860.93	€841.17
Thereof without C00–D48 diagnosis	59	€1,029.18	€717.80	51	€1,059.77	€717.80	8	€834.16	€829.96
Pleurodesis, any LOS							79	€16,478.30	**€8,867.84**
Thereof with C00–D48 diagnosis							35	€14,055.88	€8,576.23
Thereof without C00–D48 diagnosis							44	€18,405.22	€8,908.96

Bold numbers are used for further calculations. Numbers in parentheses have been amended after validation using the web grouper.

DRG, diagnosis-related group; LOS, length of stay.

#### Ascites

Cost calculation for ascites comprises a breakdown into relevant cost positions per pathway (compare Table 3).

**Table 3.** Cost Components Per Pathway (Ascites)

Ascites						
	Cost components	Drainage			Puncture	
		A1a	A1b	A2	B1	B2
Inpatient	DRG		€1,820.67		€721.54	
Outpatient	Treatment			€253.07		€15.24
	Examination					€15.9
	Material	€75.00	€75.00	€395.00		€7.07
	Implantation set			€320.00		
	Drainage kit	€75.00	€75.00	€75.00		
	Other					€7.07
	Lump sum (quarterly)					€21.80
	Home care services	€22.91	€22.91	€22.91		
Total one-off costs			€1,820.67	€573.07	€721.54	
Total variable costs		€97.91	€97.91	€97.91		€60.02
Total costs (for one procedure)		€97.91	€1,918.58	€670.98	€721.54	€60.02

A1a = inpatient implantation (intervention as the secondary reason)+homecare drainage, A1b = inpatient implantation (intervention as the primary reason)+homecare drainage, A2 = outpatient implantation, B1 = inpatient puncture, B2 = outpatient puncture.

Starting with pathway A1a, costs for the inpatient implantation have been determined to be €0 since the procedure was captured as part of a wider reimbursement code. From the German SHI perspective, cases assigned to A1a incur the same costs, independent of treating a patient with ICS or not because the particular case mix calculation is not sensitive to the addition of the “ICS-OPS” (as is often the case with more expensive DRGs).

In pathway A1b (inpatient implantation as the primary reason for hospital stay), 21 cases fulfilled the criteria. Their median DRG costs amounted to €3,286.19, but after validation using the web grouper, a less costly DRG (Z01C) was identified. Thus, we analyzed all cases with this DRG in our RWD (*n* = 18) and obtained median costs of €1,820.67, which we used in the following analysis. In both pathways, patients proceed with home care drainage, which has been quantified by summing up costs for the ICS drainage material and costs for ambulatory care service. In total, this accounts for €97.91, leading to total costs of €97.91 and €1,918.58 for A1a and A1b, respectively.

Pathway A2 (outpatient implantation for ascites followed by home care drainage) accounted for €670.98 for the initial treatment procedure. It includes material costs for the ICS and costs for the outpatient surgery reflected by the *EBM* position 5–549.2 (implantation of an ICS in the abdomen: for ascites drainage), followed by frequent drainage at home.

The subgroup “inpatient puncture” (B1) consisted of 116 patients with median costs per case of €721.54. Our web grouper validation supported this result.

Cost components that have been quantified for pathway B2 (outpatient puncture) comprise treatment, examination, and material costs, as well as the quarterly lump sum. The total costs amount to €60.02.

#### Pleural effusion

The resulting costs for pleural effusion have been determined analogously to the calculation of ascites costs and are summarized in Table 4.

**Table 4.** Cost Components Per Pathway (Pleural Effusion)

Pleural effusion						
	Cost components	Drainage		Puncture		Pleurodesis
		A1a	A1b	B1	B2	C1
Inpatient	DRG		€2,149.67	€833.70		€8,867.84 €
Outpatient	Treatment				€28.92	
	Examination				€12.24	
	Material	€75.00	€75.00		€7.07	
	Implantation set					
	Drainage kit	€75.00	€75.00			
	Other				€7.07	
	Lump sum (quarterly)				€21.80	
	Home care services	€22.91	€22.91			
Total one-off costs			€2,149.67			€8,867.84
Total variable costs		€97.91	€97.91	€833.70	€70.03	
Total costs (for one procedure)		€97.91	€2,247.58	€833.70	€70.03	€8,867.84

A1a = inpatient implantation (intervention as the secondary reason)+homecare drainage, A1b = inpatient implantation (intervention as the primary reason)+homecare drainage, B1 = inpatient puncture, B2 = outpatient puncture, C1 = pleurodesis.

Thirty-three RWD cases fulfilled the criteria for subgroup A1b (inpatient implantation). Their median DRG costs amounted to €3,263.75, but after validation using the web grouper, a less costly DRG (E73B) was identified. Thus, we analyzed all cases with this DRG in our RWD (*n* = 46) and obtained median costs of €2,149.67, which we used in the following analysis.

Implantation costs for a secondary treatment (A1a), again, amounted to €0 (A1a). The subsequent home care drainage cost was €97.91 in pathway A1a as well as in pathway A1b, resulting in total costs of €97.91 and €2,247.58, respectively.

The RWD analysis of respective pleural effusion cases who were treated with puncture (B1) indicated median DRG costs per case of €833.70 (*n* = 22), which could be validated using the web grouper.

For an outpatient puncture (B2), the SHI reimburses costs amounting to €70.03, consisting of costs for the puncture itself, the quarterly lump sum, sonography, and materials.

Based on the RWD of the UHC, patients who received pleurodesis (C1) have been identified (*n* = 79). The median DRG remuneration was €8,867.84. As pleurodeses are ideally only performed once and not regularly, this pathway only incurs nonrecurring costs.

#### Comparison of pathways (break-even analysis)

For a graphical visualization and comparison of costs resulting from different pathways, we derived cost functions based on the frequencies of each procedure (see Table 5).

**Table 5.** Frequencies of Procedures (Ascites and Pleural Effusion)

Frequencies	Ascites^24^	Pleural effusion^40,42–44^
Puncture	Every 10 days = 0.714/week	Every 10 days = 0.714/week
Implantation of ICS	One-off	One-off
Examination	Quarterly = 0.077/ week	Quarterly = 0.077/week
Home care drainage	3 Times/week	3 Times/week
Home care service performing the drainage	3 Times/week	
Pleurodesis	Not applicable	One-off
Outpatient lump sum	Quarterly = 0.077/week	Quarterly = 0.077/week

ICSs, indwelling catheter systems.

The following cost functions have been calculated for ascites and pleural effusion:

For ascites:

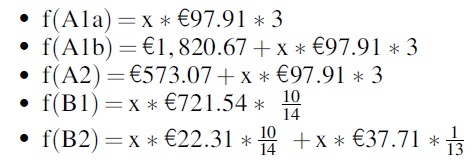

For pleural effusion:

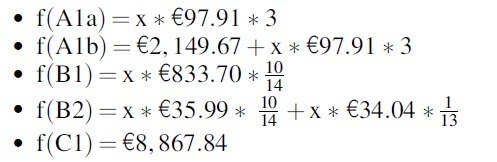

**FIG. 3.**
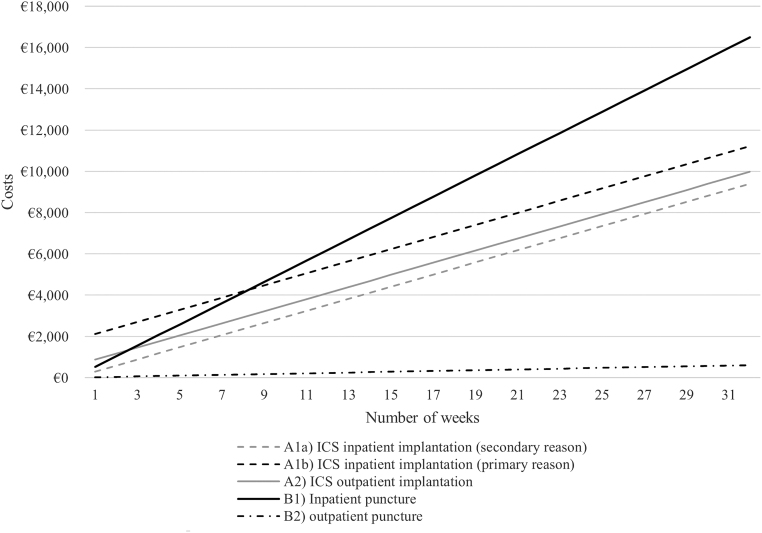
Costs per treatment pathway over time (ascites).

**FIG. 4.**
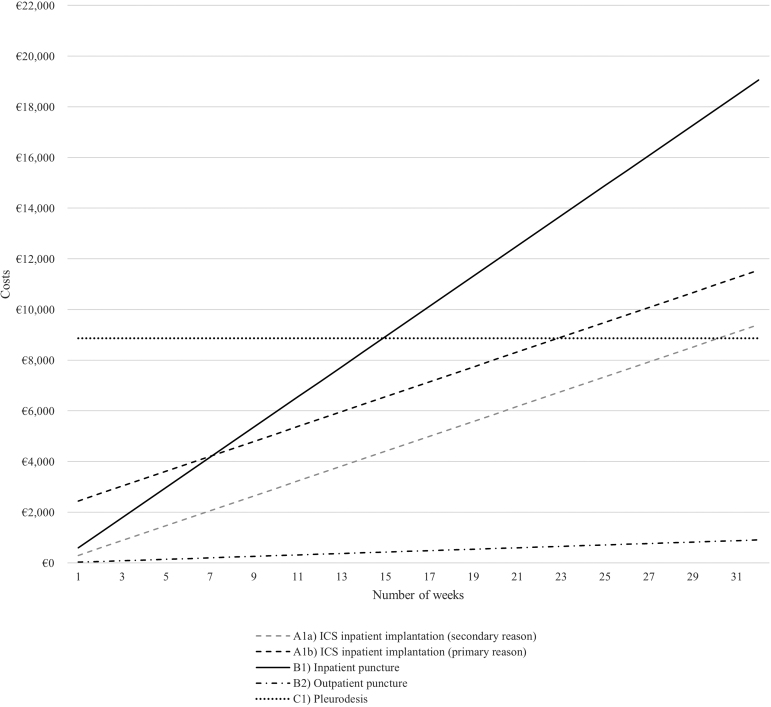
Costs per treatment pathway over time (pleural effusion).

Based on these functions, we created the referring graphical representation (Figs. 3 and 4).

Looking only at the costs for a single procedure (i.e., costs that occur in week 1), treatment costs for all pathways can be ranked (lowest to highest costs) as follows:

(1). (B2) Outpatient puncture
(2). (A1a) Inpatient ICS implantation (secondary reason)
(3). (B1) Inpatient puncture
(4). (A2) Outpatient ICS implantation (only for ascites cases)
(5). (A1b) Inpatient ICS implantation (primary reason)
(6). (C1) Pleurodesis (only for pleural effusion cases)

The relationships change when analyzing costs over the course of time. Outpatient punctures, predictably, remain the most inexpensive option. Whereas costs for inpatient punctures are relatively low in the short term, costs for all pathways incurring high one-off costs at the beginning will amortize after a period of time, turning them into economically favorable options in the medium and long term. The exact point of time when relationships change depends on the examined comparator treatments.

Inpatient treatment with ICS (primary reason; A1b), for example, will save costs compared with inpatient punctures when used for more than 8.2 weeks (ascites) and 7.1 weeks (pleural effusion). Figures 3 and 4 graphically illustrate all break-even points (i.e., intersection points of two graphs).

### Transportation costs

The share of cases with care service of stages 3 to 5 according to the German classification system amounted to 12.17% of ascites and 11.27% of pleural effusion patients. Based on common market fees, we assumed that a patient's transport to and from the physician's office results in costs of €300 in total. The SHI covers the costs, except for a copayment of €10 per drive. Thus, from a payer perspective, costs of €280 are incurred per patient entitled to patient transport.

Combining costs and the percentage of applicable ascites and pleurodesis patients, average costs amounted to €34.08 and €31.54, respectively. Thus, the total costs would increase depending on the frequency of necessary transportation per pathway, which is especially high for outpatient puncture treatment pathways.

## Discussion and Conclusions

The results of this analysis demonstrate that there are diverging cost-effective management strategies for recurrent ascites and pleural effusion. Both diseases are associated with high symptom burden for patients and poor quality of life.^17,24^ Regarding ascites, outpatient puncture has been identified as the least costly per procedure, annual quarter, and year. On the other hand, ICS when implemented as the primary reason for hospital admission was the most expensive per procedure, while inpatient punctures were the most expensive per annual quarter and year.

Similarly, for pleural effusions, outpatient punctures were found to be the least expensive per procedure, annual quarter, and year. Pleurodesis was the most expensive per procedure and annual quarter, while inpatient puncture was the costliest per year. Similar to the literature, we found that ICS for ascites brings a cost advantage in comparison with inpatient large-volume paracentesis for patients after 2.6 weeks when implemented in an outpatient setting and after 8.2 weeks when implemented in an inpatient setting.^19,20^

Regarding pleural effusion, we identified that the cost of pleurodesis was higher compared with ICS until 30.2 weeks (secondary reason; A1a) and 22.9 weeks (primary reason; A1b) of treatment. Our results are in line with results derived in the study by Botana-Rial et al. who identified a slightly shorter time interval.^21^ Several studies indicate that life expectancy in patients with malignant pleural effusion can range from 3 to 12 months.^25,26^ In patients with end-stage liver disease with refractory ascites, life expectancy is, on average, six months.^27^

The present study compared the costs of different management therapies for ascites and pleurodesis. If either disease occurs, an occurrence is more likely toward the end of life, which is an indicator of poor outcomes. Pleural effusion affects the quality of life and is a significant cause of dyspnea and patient suffering. Puncture and drainage provide symptomatic relief. Pleurodesis and ICS share a similar success rate for controlling malignant pleural effusion in terms of survival and patient-reported dyspnea.

On the one hand, the risk of cellulitis was higher in the ICS group, but on the other hand, the implementation of a catheter shows a reduced inpatient LOS.^28,29^ Furthermore, patients with malignant pleural effusions receiving an ICS show fewer hospital readmissions and require fewer pleural procedures in comparison with patients with pleurodesis.^12,29–33^ Especially malignant and paramalignant effusions into the pleural space are associated with poor outcomes.^34^ These patients therefore may be better suited for symptomatic treatment using ICS rather than pleurodesis.

Our analysis identified puncture as both the least and most expensive therapy option depending on outpatient or inpatient treatment. However, determined by the individual patient's situations, it is likely that patients receive the treatment in both settings over the course of their treatment. These circumstances indicate that the modeled cost approach presented in this study is a simplification of the complex reality of possible treatment alternatives. In the context of diverging treatment options such as ICS or puncture for ascites and pleural effusion, potential complications should also be discussed.

The most common complications of abdominal paracentesis include ascitic fluid leakage, hemorrhage, infection, and drainage of protein and electrolytes, as well as bowel perforation.^35,36^ In pleural effusion, the most common complications associated with catheter placement involve subcutaneous and pleural infections, potential catheter rupture, and hemorrhage.^25,37^

Among patients with malignant pleural effusion, pleurodesis failure rates are known to be ∼20% to 25% over the subsequent three months.^38^ In consequence, these patients are readmitted, resulting in increased treatment costs. Consideration of the individual circumstances of the patient (stage of disease, prognosis, and bleeding disorder) is also necessary for treatment selection to minimize the risk of aforesaid complications.

### Implications for palliative care

Home-centered treatment options such as drainage can reduce readmission rates and improve the quality of life of patients. In this way, patients spend less time in hospitals or outpatient clinics, hence lowering the risk of hospital-associated infections and increasing patient safety. Studies have shown that treatment at home is usually preferred by patients at end of life, thus also leading to a gain in quality of life. In addition to avoiding hospitalizations and readmissions, a catheter can also lead to significant improvement in overall health and relief of symptoms.

It also offers ease of use according to home care nurses. Several authors have demonstrated successful treatment of malignant pleural effusion with the PleurX catheter—a system that can, however, become costly over time.^18,39,40^ The ASEPT pleural catheter appears to be comparable and, according to Dhaliwal et al., may ultimately provide cost savings through advantages in design.^39^

### Limitations

Although we applied the best health economic standards, our study has some limitations. First, the RWD were collected from a single academic institution, thus reducing the results' generalizability and external validity. However, a large sample of over 4000 cases has been analyzed. Second, both in- and outpatient therapy options were included; however, distribution to the groups was not randomized. This leads to various biases. Third, outpatient treatment was simulated. Fourth, certain assumptions had to be made, for example, transaction costs for patients traveling to hospitals and that home care was provided in all ICS cases.

Fifth, we were not able to analyze individual patient journeys, for example, from development of symptoms to death, and were not able to identify the reasons for the LOS. Nevertheless, with the help of simulations, an economic evaluation was performed from a metaperspective, allowing a general comparison of underlying treatment options. Finally, another limitation is that we were not able to include data concerning (potentially cost-driving) complications and overall medical institution visits of individual patients.

## Conclusions

In conclusion, the present study is one of the first German cost studies on this topic, paving the way for future research. We suggest that future research should collect and analyze RWD on clinical and patient-reported outcomes—in particular, EQ-5D-5L data—to provide a more holistic health economic perspective. However, there are challenges involved in conducting clinical trials in a palliative setting, including, but not limited to, the definition of the palliative phase, recruitment, increased attrition, and uncertainty.^24,41^

Considering demographic changes, disruptions in financing of the health system, and a shift toward the outpatient setting, it is important to continuously assess whether ICSs can contain costs with better outcomes for patients. Our study paves the way for this.
